# Supplementary material for: SHARPER-DOSY: Sensitivity enhanced diffusion-ordered NMR spectroscopy
Source: Nat Commun. 2023 Jul 21;14:4410. doi: 10.1038/s41467-023-40130-2 (PMC10361965; doi:10.1038/s41467-023-40130-2)
Supplement: Supplementary file 2 — Supplementary Information [file 41467_2023_40130_MOESM2_ESM.pdf]

Supplementary information

SHARPER-DOSY: Sensitivity Enhanced Diffusion-Ordered NMR Spectroscopy

George Peat,[1] Patrick J. Boaler,[1] Claire L. Dickson,[1,2], Guy C. Lloyd-Jones,[1] Dušan Uhrín*[1]

Table of Contents

Supplementary Note 1: Annotated pulse sequences ……………………………………………………………...2
Supplementary Note 2: Analysis of the time domain data and spectra of **1**……………………………………...........3
Supplementary Note 3: Shape of the SHARPER signal………………………………………………................5
Supplementary Note 4: Comparison of signal intensities of 1D ^{1}H and SHARPER spectra of **1**……………….5
Supplementary Note 5: Removal of the imaginary part of SHARPER time domain points...............................5
Supplementary Note 6: Compensating for the magnetic field inhomogeneity by SHARPER……………………6
Supplementary Note 7: Power deposition during SHARPER acquisition……………………………………....6
Supplementary Note 8: Multi-resonance suppression of unwanted signals by phase modulated low power rectangular pulses…………………………………………………………………………………………………..7
Supplementary Note 9: 1D ^{1}H and SHARPER spectra of **2**……………………………………………………....10
Supplementary Note 10: Time domains or spectra? Analysis of diffusion coefficients…………………………12
Supplementary references ………………………………………………………………………………………13

Supplementary Note 1: Annotated pulse sequences

1.1 Pulse sequence of non-selective SHARPER

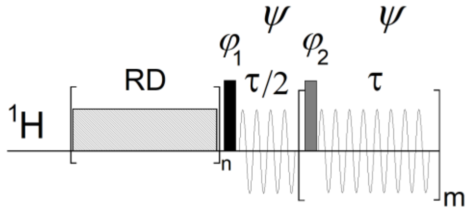

Supplementary Fig. 1.1. Pulse sequence of a non-selective SHARPER experiment with a presaturation module. A black filled square represents a 90° non-selective pulse, while a reduced power grey pulse of the SHARPER module can have arbitrary flip angle (180° or 90° recommended). RD – relaxation delay with signal saturation, τ - acquisition chunk time. The following phases were used: φ_1=2x, 2(-x); φ_2=y,-y; Ψ = 2x, 2(-x). n – a number of repetitions of the saturation pulse, m – number of repetitions of the pulse, acquisition chunk blocks. The corresponding Bruker pulse programmes **sharper_collapse** (see Supplementary Software).

1.2. Selective excitation by Band Selective Perfect Echo (BSPE) and BSPE-SHARPER

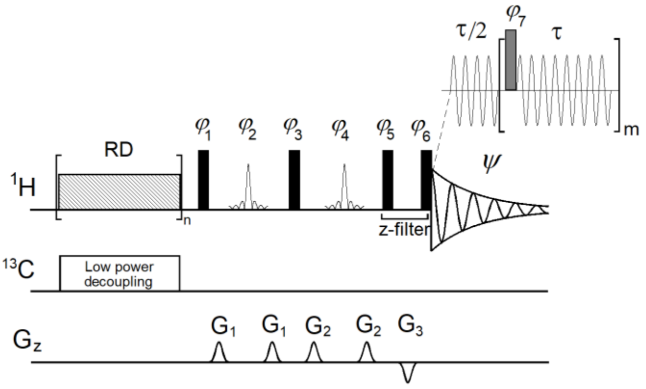

Supplementary Fig. 1.2. Pulse sequence o BSPE and BSPE-SHARPER (top acquisition scheme) with a presaturation module and optional z-filter. Black filled squares represent 90° non-selective pulses, while a reduced power grey pulse of the SHARPER module can have arbitrary flip angle (180° or 90° recommended). 180° band selective ReBurp pulses are applied in the middle of the BSPE. The delays between pulses are limited to PFG and the recovery delay. RD – relaxation delay, τ - acquisition chunk time. The phases of the BSPE experiment are: φ_1=4x, 4(-x); φ_2=8y, 8(-y); φ_3=2y, 2(-y); φ_4=8y, 8(-y); φ_5=-x, φ_6=x, φ_7=y,-y, and Ψ = 4x, 4(-x). The pulsed field gradients were set to: G_1 = 7%, G_2 = 5%, G_3 = -12%. The corresponding Bruker pulse programmes, **zgbspe** and **sharper_collapse** (see Supplementary Software).

1.3. Pulse sequence of SHARPER-DOSY

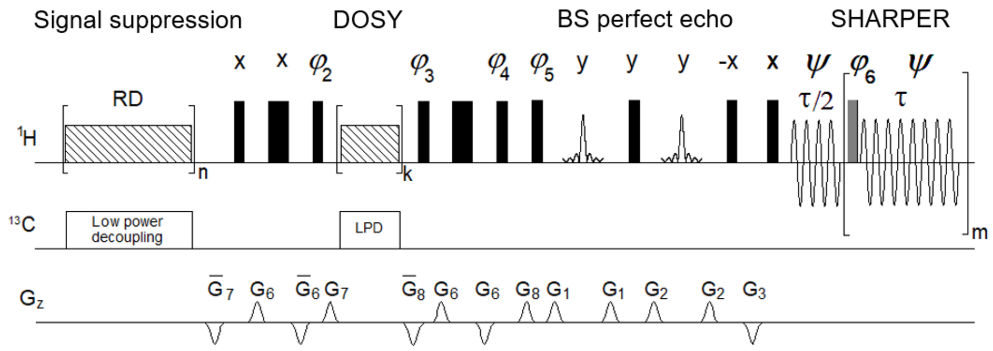

Supplementary Fig. 1.3. Pulse sequence of the SHARPER-DOSY experiment with optional presaturation module, band-selective perfect echo and a z-filter. Black narrow and wide filled squares represent 90° and 180° non-selective pulses, while a reduced power grey pulse of the SHARPER module can have arbitrary flip angle (180° or 90° recommended). 180° band selective ReBurp pulses are applied in the middle of the BSPE. The delays between pulses are limited to PFG and the recovery delay.). RD – relaxation delay, τ - acquisition chunk time, n,m,k represent the number of loops. The phases are: φ_2=2x, 2(-x); φ_3=4x, 4(-x); φ_4=2(x,-x), 2(-x, x); φ_5=4x, 4(-x); φ_6= 8y, 8(-y) Ψ = x, 2(-x), x, -x, 2x,-x.The gradients strength as a % of the total value (66.4 G/cm) are G_1 = 7%, G_2 = 5%, G_3 = -12%, G_6 = 5 to 95%, G_7 = -17.13%, G_8 = -13.17%. The corresponding Bruker pulse program **ledbpgp2s.sharper_collapse** (see Supplementary Software).

Supplementary Note 2: Analysis of the time domain data and spectra of 1

The time domain points of NMR experiments, in which the signal is manipulated during acquisition, are acquired effortlessly by Bruker Topspin pulse programs. Data acquisition is paused at the end of each data chunk and restarted after a block of pulses and delays. The build-up of the signal can be viewed in real time on a computer screen. No data manipulation is required, chunks of data points are arranged to form an "FID" that can be processed in a regular manner in Topspin or any other NMR processing software.

The time domain data corresponding to the spectrum of **1** presented in Fig. 2 of the main paper is analysed here in more detail. Figures S2.1a and b show the real an imaginary parts time domain points acquired using the pulse sequence of Fig. 2a, dwell time of 50μs and 4 data points (i. e. 2 complex points) during a 200μs chunk time, τ. The decaying signal was directed into the real channel by adjusting the phase of the receiver. A slow modulation of the time domain points with a period of 0.384s visible in both channels remains unexplained; nevertheless, it did not manifest itself in the corresponding spectra shown in Supplementary Fig. 2.2. Supplementary Fig. 2.1c shows 200 real points acquired over 20 ms with the initial group delay points of the Bruker Avance digital filter removed (these points do not contain any spectral information). The points acquired during the first few echoes show intensity fluctuations, which settle quickly.[1] Supplementary Fig. 2.1d shows an expansion containing 10 real points acquired over a period of 1 ms. These show the expected modulation with the frequency equal to the inverse of the chunk time producing sidebands at ± 1/τ (1/200e-6) = ± 5000 Hz. No intensity variations that could be attributed to the insertion of 60μs ^{1}H pulses between individual data chunks are visible. This is due to long effective relaxation of **1** during the SHARPER acquisition (T_2^S> 2.89 s, calulated as $T_2^S = 1/(\pi\Delta_{1/2})$, where $\Delta_{1/2} = 0.11\ Hz$).

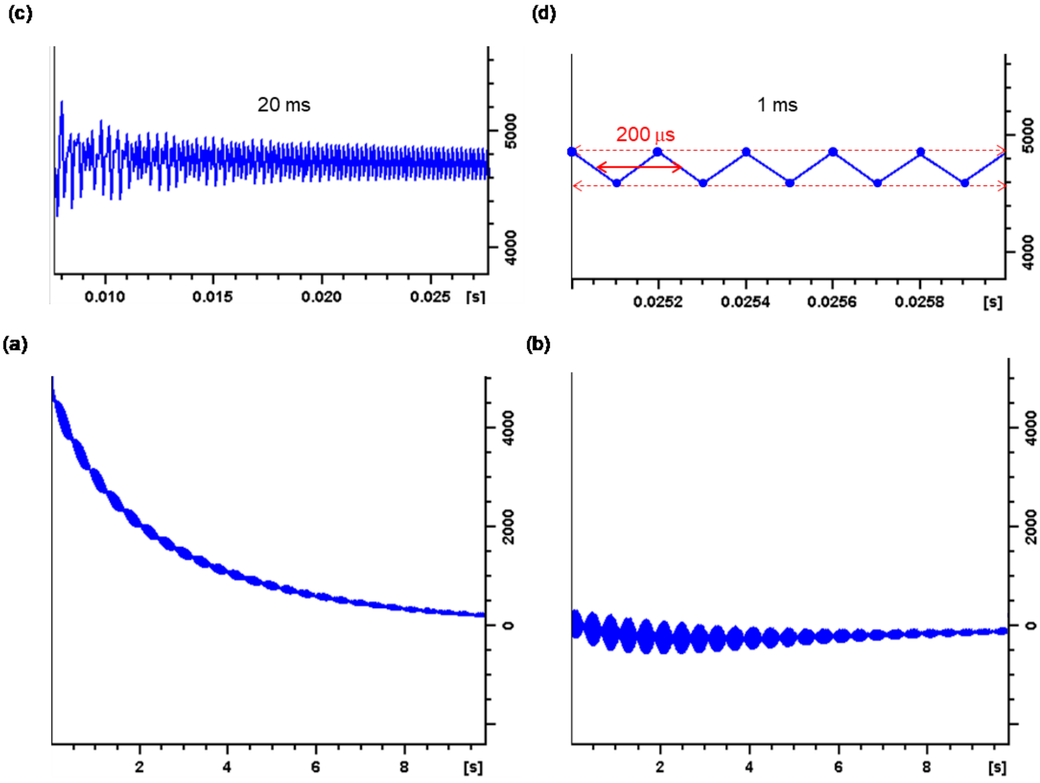

Supplementary Fig. 2.1. 400 MHz ^{1}H SHARPER time domain data points of the spectrum of **1** presented in Fig. 2 of the main paper; (a) and (b) show the real and imaginary points, respectively. (c) 200 real points acquired over 20 ms. (d) 10 real time domain points acquired over 1 ms. Modulation of the data over one chunk period of 200 μs is highlighted. The following parameters were used: dwell time: 50μs, chunk time: τ = 200μs, and 4 data points (or 2 complex points).

A step decrease in the signal intensity is visible for fast relaxing spins, e.g. of polymers, when selective pulses of comparable length to those of chunk times (~20 ms) are used to refocus the evolution of homonuclear coupling constants[2] (Supplementary Fig. 2.2a). Nevertheless, as these discontinuities coincide with the chunk length, the artefacts they create in spectra appear at the same frequency as the chunking artefacts and do not interfere with the main signal (Supplementary Fig. 2.2d). Whether short or long r.f. pulses are used, these contribute towards the overall T_2^S relaxation, shortening it and broadening the SHARPER singlet. For long selective pulses this

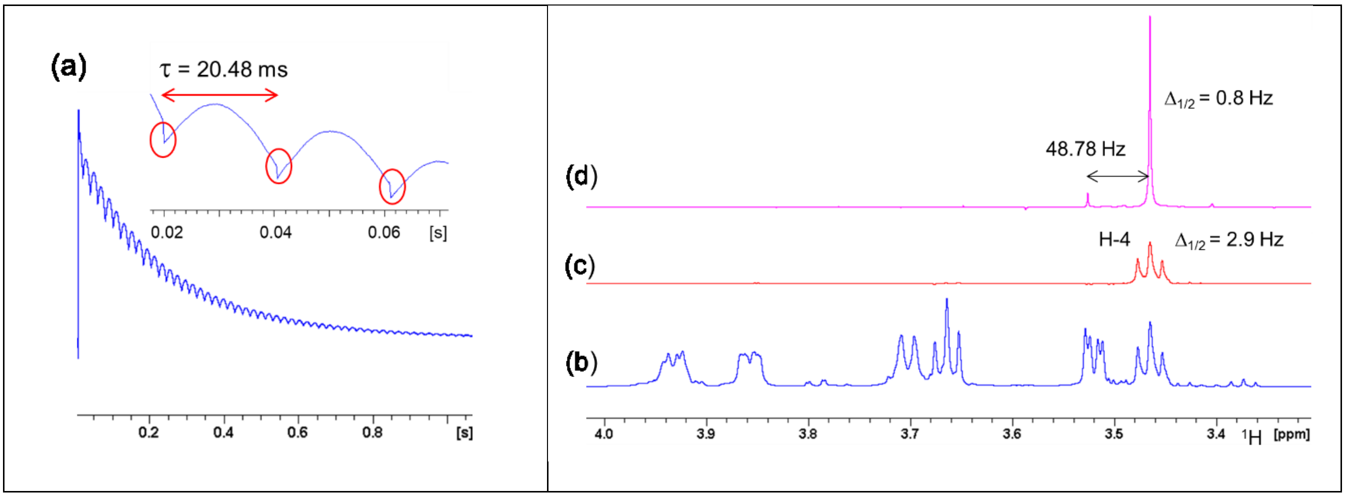

Supplementary Fig. 2.2. 800 MHz ^{1}H NMR data and spectra of dextran (Mw=5.6 kD). (a) The real data points of SHARPER acquisition of H-4. The insets show a 50ms expansion with the chunk time, τ, indicated. A partial evolution due to *J* couplings takes place and a drop of signal intensity (circled) caused by 20 ms selective Gaussian pulses interrupting the acquisition, is visible. (b) ^{1}H NMR spectrum of dextran. (c) A chemical-shift-selective filter (CSSF) spectrum selecting H4 using a 40 ms Gaussian pulse and eight 1.4 ms increments of the filter (d) A SHARPER-CSSF spectrum of dextran with the sideband frequency and the half widths of the signal stated. All spectra were acquired in 16 scans using identical parameter. Considerable signal enhancement is visible in (c), while the main SHARPER signal is not influenced by the modulations and discontinuities of the raw data.

Returning to the domain data of **1,** the spectra obtained by Fourier transformation of the data points from both channels and by using only the real data points are shown in Supplementary Fig. 2.3a, b and c, d, e, respectively. The latter show reduced noise over the entire frequency range and in particular around the central peak (compare the insets on the right in Supplementary Fig. 2.3a and c). The intensity of the chunking sidebands that are positioned just below ± 5000 Hz (= $1/\tau$ = 1/(200e-6) Hz) is reduced when only the real data points are used (compare the insets in Supplementary Fig. 2.3b and d); their intensity is at ~ 3% of the main SHARPER peak. The resonance frequency of the sidebands is slightly lower than 5000 Hz due the additional delays of the order of nanoseconds, which are inserted into the spin-echoes to allow the on/off switching of the receiver. The low level artefacts in the ± (2000-3000) Hz regions cannot be fully explained; their broader components can be removed by applying a backward linear prediction (see Supplementary Fig. 2.3e). Overall, the SHARPER spectra of **1** are very clean; the intensity of the described minor signals is low and inconsequential for the quantification of the SHARPER signal, which sits in the clean central region of the spectrum.

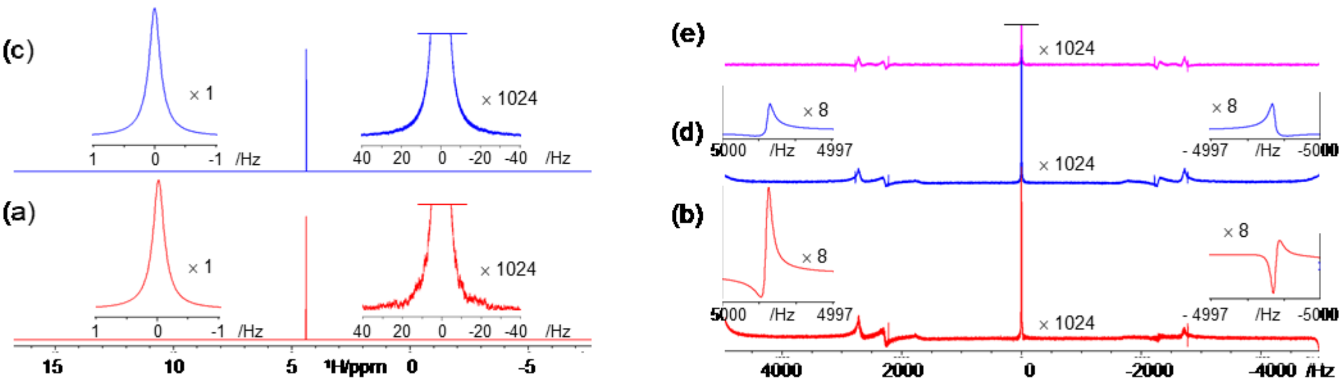

Supplementary Fig. 2.3. 400 MHz ^{1}H SHARPER spectra of **1** (see also Fig. 2 of the main paper) produced by processing of the time domain data shown in Supplementary Fig. 2.1. In (a) and (b), both the real and imaginary data points were used and a line broadening of 0.11 Hz (a matched filter) applied; In (c), (d) and (e) only the real points were used. The insets in (a) and (c) show vertical expansions of the SHARPER signal over ± 1 and ± 40 Hz, respectively. (b) and (d) show a 1024-fold vertical expansion of (a) and (c). Here the insets show 8-fold vertical expansions of the areas around the first sidebands. (e) The same as (d) but with a linear backward prediction of the first 100 points applied to real time domain points only. For comments see the text.

Supplementary Note 3: Shape of the SHARPER signal

Fourier transformation of a SHARPER signal of a single nucleus decaying with an effective relaxation time T_2^S produces an absorption Lorentzian line (Eqn. 1):

$$S(\upsilon) = \frac{1/T_2^S}{(1/T_2^S)^2 + 4\pi^2(\nu - \nu_L)^2} \qquad (1)$$

with maximum intensity at $\nu = \nu_L$ equal to T_2^S, where ν_L is the Larmor freqeuncy of the SHARPER signal. The width of this Lorentzian line at the half height, $\Delta_{1/2}^S$, is given by Eqn. 2

$$\Delta_{1/2}^S = (1/\pi T_2^S) \qquad (2)$$

The intensity of the Lorentzian line therefore is proportioned to T_2^S and inversely proportioned to $\Delta_{1/2}^S$. When comparing SHARPER signals acquired under different conditions, it is convenient to determine their half widths, calculate T_2^S (Eqn. 2) and use this value as a factor in determining the signal intensity. When collapsing spectra containing protons with different spin-spin relaxation times, the SHARPER signal is not a pure Lorentzian line, nevertheless, using this procedure still provides a reasonable representation of its height.

Supplementary Note 4: Comparison of signal intensities of 1D ^1H and SHARPER spectra of 1

Without any apodisation, the intensity ratio of the SHARPER signal *vs* the CH_3 signal from the 1D ^1H spectrum is given by Eqn. 3.

$$Intensity\ ratio = \left(\frac{9*2}{3}\right) * \frac{T_2^S}{T_{2eff}^{CH_3}} * collaps\ efficieny = 6 * \frac{1.372}{0.289} * 0.87 = 24.7, \qquad (3)$$

where the initial factor accounts for different number of protons contributing to each signal and the fact that the methyl is a doublet. The ratio of T_2^S values quantifies the narrowing effect of SHARPER acquisition and 0.87 is the efficiency of collapsing signals of individual protons of **1** based on their number and the distance from the carrier frequency as shown in Fig. 3a calculated as a weighted sum of integrals. The results are in perfect agreement with the experimental data shown in Fig. 2.

Supplementary Note 5: Removal of the imaginary part of SHARPER time domain points (zim.py)

The signal-to-noise ratio (SNR) in the 1D spectrum of **1** and its SHARPER spectrum was maximised independently by applying match exponential filters[3] (line broadening, $LB = \Delta_{1/2}$ = 0.59 and 0.11 Hz, respectively), producing an 8.3-fold improvement for the SHARPER spectrum. Note that the ratio of signal intensities between the two spectra remains the same, as applying a matched filter halves the T_2^S values of both signals. A larger *LB* value used in processing of the 1D ^1H spectrum, results in a larger noise reduction. As demonstrated previously,[4] by removing the imaginary time domain points, additional gain of √2 in SNR is obtained, producing the final stated 11.4-fold increase.

A python script (zero-imaginary, **zim.py**), provided in Supplementary Software, zeroes the imaginary data points of Bruker 1D or 2D data. The script requires the numpy, scipy and nmrglue packages; these packages can be installed using, for example, **(python -m) pip install nmrglue**. A convenient starting point for this is the anaconda python distribution, which installs python alongside current versions of the numpy and scipy packages (among many others).

The **zim.py** script can be run from the terminal and returns the modified Bruker data as a separate directory in which the original name has been appended with "-zim". The script comes with two modes, one for performing the operation on a particular experiment (using the optional **--expno** followed by an integer) and one for performing the operation on all experiments within a directory whose pulse programmes contain the string of characters "sharp" (not case sensitive), the final argument in all cases is the path to the directory which contains all of the experiments. A user guide can be accessed by running **python zim.py --help** in the terminal.

In the following example, opening the anaconda prompt (windows) and typing:

python zim.py --expno 1 "C:\nmr\project\sample_data" will create a new directory "C:\nmr\project\sample_data-zim" containing only one subdirectory, for experiment 1. If no experiment corresponding to the number entered is found, the script will return only an OsError statement.

Similarly, running **python zim.py "C:\nmr\project\sample_data"** will return a directory "C:\nmr\project\sample_data-zim" containing processed datasets corresponding to all of the experiments in the source directory for which PULPROG contains "sharp".

In the development of this **zim.py**, the anaconda distribution of python was used with the language and package versions: python 3.7.11, numpy 1.21.5, scipy 1.7.3 and nmrglue 0.9.

Supplementary Note 6: Compensating for the

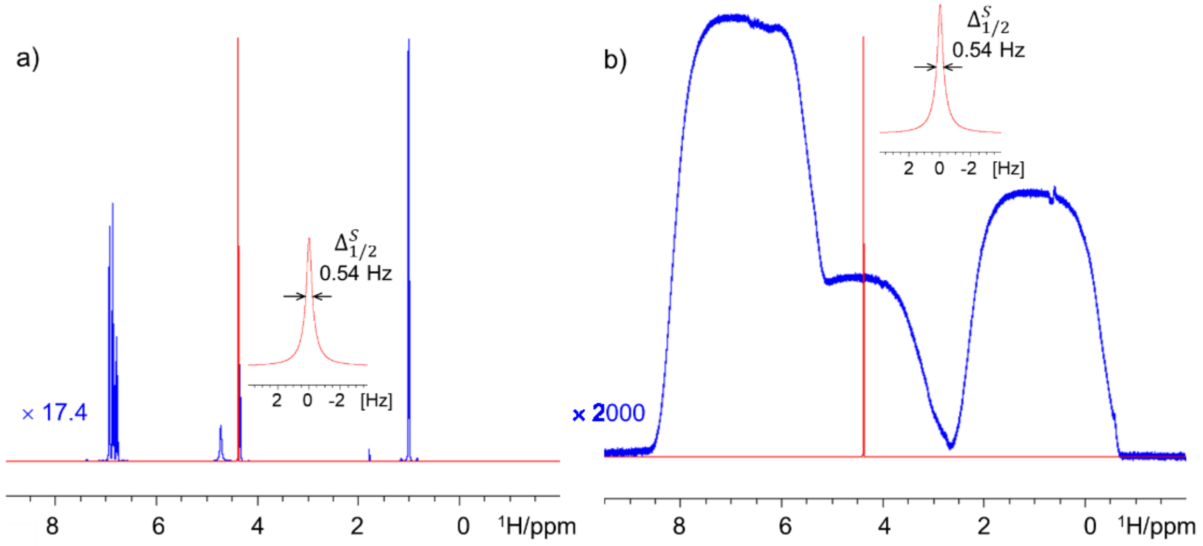

Supplementary Fig. 6.1. Overlays of 400 MHz 1D 1H NMR spectra of a neat sample of 1 and the corresponding SHARPER spectra acquired using chunk time, τ = 200μs. (a) well shimmed sample, (b) the z shim correction offset by 10,000 units relative its optimal value. No window function was applied. The vertical intensity of the 1D spectrum in (a) was scaled up 17.4-fold to place the CH3 signals on the level of the SHARPER singlet; in (b) the scaling factor was 2000. As seen in the insets, the half width of the SHARPER signals is identical regardless of the conditions.

Supplementary Note 7: Power deposition during SHARPER acquisition

Acquisition of SHARPER signals could extend beyond 10 s, hence careful consideration of power deposition is required. Bruker specification of the permitted value of CW irradiation on the room temperature probe used in this study was 5 W, corresponding to 30 μs 90°pulse. Specifications are more stringent for cryoprobes; depending on the vintage, power levels generating 80 or 100 μs 90°pulses are permitted. We have adhered to these cryoprobe specs for τ = 50 μs. As acquisition chunks (τ) separate pulses during SHARPER acquisition, for chunk times of 100 and 200 μs, a power level corresponding to 40 μs 90°pulses was used. For τ = 400 μs we used power level corresponding to twice the pulse length of a calibrated full power nonselective pulse (adding 6 dB). We have not observed any heating on non-lossy samples. When 90° spin-echo pulses are used, power deposition is reduced two-fold. Although some loss of integral intensity occurs (Supplementary Fig. 7.1a), this is compensated for at least partially by the increased signal intensity (Supplementary Fig. 7.1b).

Another parameter that must be considered in CPMG experiments is the ringdown time. Due to the high Q factor of cryoprobes probes, the ring-down time of the RF coil after short pulses is typically longer than on the room temperature probes and could vary between few microseconds to hundreds of microseconds. In our experience, a 2 scan phase cycle of the spin-echo pulses (90°, 270°) or any 2 scan phase cycle of within the pulse sequence that necessitates an inversion of the receiver phase eliminates any artifacts caused by the leakage of the r.f. pulses, however, caution must be exercised not to overload the preamplifier.

We strongly encourage potential user of this techniques to consult the manufacturer of their spectrometer for recommended pulse power levels and ringdown times; these are probe specific.

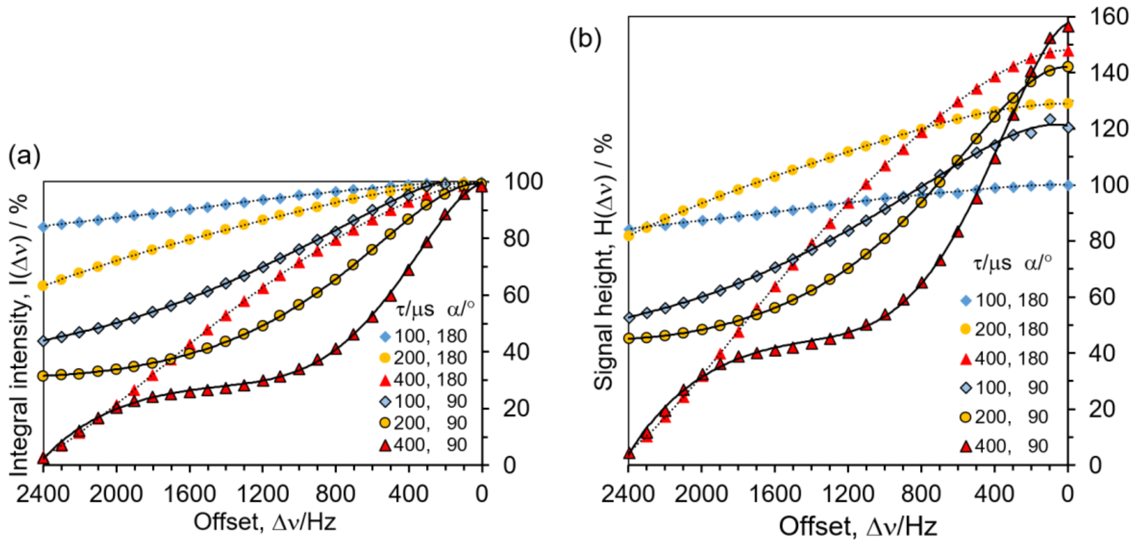

Supplementary Fig. 7.1. Efficiency of the collapsing of NMR spectra into a singlet. (a) Relative integral intensity, $I(\Delta\nu)/I(0, \tau = 100\mu s)$, and (b) relative signal height, $H(\Delta\nu) = T_2^S \times I(\Delta\nu)/(T_2^S(\tau = 100\mu s) \times I(0, \tau = 100\mu s))$, profiles for SHARPER singlets of HOD in a doped D_2O sample at 400 MHz as a function of the frequency offset, Δν. The intensity (a) and the height (b) of the on-resonance signal for τ = 100 was normalised to 100. The key shows the length of the chunk time, τ, and the spin-echo pulse angle, α. Experimental parameters are given in the Methods (main paper). Source data are provided as a Source Data Supplementary Fig. 7.1.xlsx.

Supplementary Note 8: Multi-resonance suppression of unwanted signals by phase modulated low power rectangular pulses

8.1. Calculating the length of the selective pulse – fixed carrier frequency (Multi-reson-suppress.xlsx).

In the rotating frame, off resonance signals precess around a magnetic field according to Eqn 4,

$$\Delta\nu = \frac{\gamma\Delta B}{2\pi} \qquad (4)$$

where, ΔB is the residual magnetic field and $\Delta\nu$ is the rotating frame frequency. The angle of precession around the field of an off-resonance signal during a pulse length τ is calculated as

$$\theta = \gamma\Delta B\tau \qquad (5)$$

Hence, the number of rotations (x) of this signal is given as

$$\theta = 2\pi x \qquad (6)$$

Substituting and solving for $\gamma\Delta B$ gives

$$\gamma\Delta B = \frac{2\pi x}{\tau} \qquad (7)$$

Substituting Eqn 4 into Eqn 7 and taking x as an absolute value gives

$$x = \tau\,\Delta\nu \qquad (8)$$

The x and $\Delta\nu$ components of Eqn 8 can be generalised as vectors $\mathbf{x}$ and $\boldsymbol{\Delta\nu}$

$$\mathbf{x} = \tau\,\boldsymbol{\Delta\nu} \quad \text{where} \quad \mathbf{x}, \boldsymbol{\Delta\nu} = \begin{pmatrix} x_1 \\ x_2 \\ \vdots \\ x_n \end{pmatrix}, \begin{pmatrix} \Delta\nu_1 \\ \Delta\nu_2 \\ \vdots \\ \Delta\nu_n \end{pmatrix} \qquad (9)$$

For effective presaturation of multiple signals, an integer number of rotations is required for each component ($x_n \in \mathbb{Z}$). The distance to nearest integer (DNI) function (Eqn. 10) can be applied to the vector to give the total distance of all components of the vector from an integer as a single value, referred to here as the "DNI norm" $\|\mathbf{x}\|_{DNI}$ (Eqn. 11). Minimising this norm by varying τ gives an optimised pulse length, which will often be very close to integer rotations in all components.

$$DNI(x) = \min\{|x - m| \mid m \in \mathbb{Z}\} \qquad (10)$$

$$\|\mathbf{x}\|_{DNI} = \sum_i DNI(x_i) \qquad (11)$$

The optimal pulse-length τ_{opt} is then given by Eqn. 12.

$$\tau_{opt} = \min\|\tau\ \mathbf{\Delta\nu}\|_{DNI} \qquad (12)$$

Because the DNI norm surface of $\mathbf{\Delta\nu}$ is rough, minimisation using iterative methods leads to the many local minima, which often correspond to highly suboptimal presaturation parameters. Hence a brute-force method was employed to search for minima within a small range of a starting pulse-length (± 20%).

This is implemented in an Excel spreadsheet **Multi-reson-suppress.xlsx** provided in Supplementary Software. Supplementary Fig. 8.1. shows the user interface in this file. The input parameters used describe three signals at offset frequencies $\mathbf{\Delta\nu} = (573, -573, -747)^T$. This corresponds to the resonances of tetrahydrofuran and water in $CDCl_3$ at 600MHz (2256 Hz, 1110 Hz, 936 Hz) when the carrier frequency is set at 1683 Hz, directly between the two tetrahydrofuran resonances. Supplementary Fig. 8.2 shows a simulation of the DNI norm as a function of pulse-length, highlighting the difficulty of iterative minimisation.

User input				Optimiser Output			Degrees from integer number of revolutions / °	
Carrier frequency / Hz	Frequencies to suppress / Hz	Pulse length / µs		Pulse Length / µs	57590.4		Resonance 1	0.252288
1683	2256	70000	--->	DNI norm	0.0214304	--->	Resonance 2	0.252288
	1110						Resonance 3	7.210368
	936						Resonance 4	0
	0						Resonance 5	0
	0							

N.B: By default, signals at 0 Hz are not suppressed. To enable suppression, input a value arbitrarily close to 0 (e.g. 0.0001 Hz).

Supplementary Fig. 8.1. User interface for the Excel implementation of the pulse-length optimiser routine showing optimisation of pulse-length for THF and water resonances at 600 MHz. User input is shown alongside the output of the optimiser and extent of optimisation for each of the input resonances.

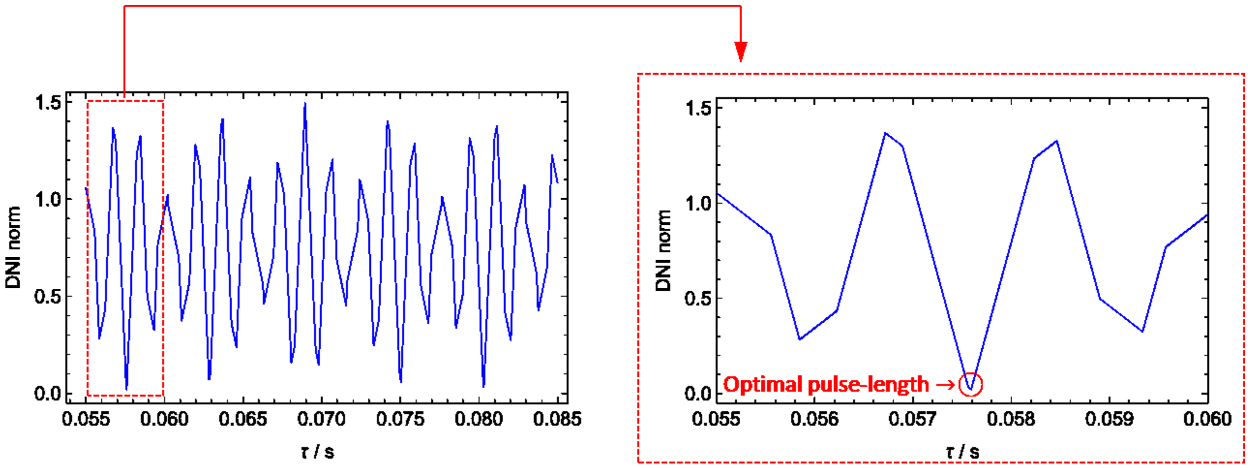

Supplementary Fig. 8.2 Simulated DNI norm $\|\mathbf{x}\|_{\mathbf{DNI}}$ (where the vector $\mathbf{x}$ is equal to $\boldsymbol{\tau}\ (573, -573, -747)^T$) as a function of pulse-length $\boldsymbol{\tau}$ between 55 ms and 85 ms; Right: simulated DNI norm for the same vector between 55 ms and 60 ms, highlighting the minimum. Source data are provided as a Source Data Supplementary Fig. 8.2.xlsx.

The multi-resonance suppression optimizer is provided as a Supplementary Software.

8.2. Calculating the length of a selective pulse – adjustable carrier frequency (PresatOptimise.jl)

In cases where the exact carrier frequency is not critical (such as in the acquisition of the collapsed SHARPER spectra), the presaturation can be further optimised by redefining the vector $\mathbf{\Delta\nu}$ as the difference between the absolute frequencies $\boldsymbol{\nu}$ and the carrier frequency ν_0. Minimisation of the DNI norm with respect to both τ and ν_0 often provides significantly improved presaturation and should be used for experiments in which the exact value of ν_0 is not critical. In these cases the optimal parameters are given by Eqn. 13.

$$\tau_{opt}\ , \nu_{0\ opt} = \min\|\tau\ (\boldsymbol{\nu} - \nu_0)\|_{DNI} \qquad (13)$$

Minimisation only with respect to τ and ν_0 was implemented in the Julia programming language (**PresatOptimise.jl**) and is available in Supplementary Software. It can be run from the command line. Installation instructions for the Julia programming language can be found at https://julialang.org. An example workflow using the Julia REPL is shown in Supplementary Fig. 8.3. The PresatOptimise function searches a 2D space around the input parameters pulse-length (τ) and carrier frequency (ν_0), where τ is allowed to vary ±20%, and ν_0 is allowed to vary ±2.5 Hz. In the example procedure shown in Supplementary Fig. 8.3, two calls to the function PresatOptimise are shown, the first starting from the original proposed carrier frequency 1683 Hz, and the second from a slightly higher frequency (1690 Hz), so that a different part of the parameter-space can be explored.

```
C:\Windows\System32\cmd.exe

C:\Users\[redacted]\Scripts>julia
               _
   _       _ _(_)_     |  Documentation: https://docs.julialang.org
  (_)     | (_) (_)    |
   _ _   _| |_  __ _   |  Type "?" for help, "]?" for Pkg help.
  | | | | | | |/ _` |  |
  | | |_| | | | (_| |  |  Version 1.7.2 (2022-02-06)
 _/ |\__'_|_|_|\__'_|  |  Official https://julialang.org/ release
|__/                   |

julia> include("PresatOptimise.jl")

--Multi-resonance presaturation parameter optimiser--

Usage: call the function PresatOptimse( [freq1 , freq2, ... ], pulse-length , carrier freq )

Units: freq -> Hz, pulse-length -> s, carrier freq -> Hz

Ensure all inputs are floating point numbers

julia> PresatOptimise([2256.,1110.,936.],0.07,1683.)

Optimised pulse length is 0.05757655765576558 s
Optimised carrier frequency is 1682.85 Hz
Optimised DNInorm is 0.018325052505261397


julia> PresatOptimise([2256.,1110.,936.],0.07,1690.)

Optimised pulse length is 0.06893729372937295 s
Optimised carrier frequency is 1690.27 Hz
Optimised DNInorm is 0.005015709570955096


julia> exit()

C:\Users\[redacted]\Scripts>
```

Supplementary Fig. 8.3. Example command prompt and REPL workflow (commands highlighted in yellow) showing the use of the Julia package "PresatOptimise.jl" for minimising with respect to both τ and ν_0, reducing the need for manual optimisation of ν_0.

The utility of this method is revealed by inputting the Julia-optimised parameters into the supporting Excel document described above. Supplementary Fig. 8.4. shows that the total extent to which the three resonances deviate from an integer number of rotations is reduced compared to Supplementary Fig. 8.1, predicting excellent presaturation performance for all three resonances.

User input		
Carrier frequency / Hz	Frequencies to suppress / Hz	Pulse length / µs
**1690.27**	**2256**	**70000**
	**1110**	
	**936**	
	**0**	
	**0**	

--->

Optimiser Output	
Pulse Length / µs	**68936**
DNI norm	**0.00597272**

--->

Degrees from integer number of revolutions / °	
Resonance 1	**0.3012192**
Resonance 2	**0.5373792**
Resonance 3	**1.3115808**
Resonance 4	**0**
Resonance 5	**0**

N.B: By default, signals at 0 Hz are not suppressed. To enable suppression, input a value arbitrarily close to 0 (e.g. 0.0001 Hz).

Supplementary Fig. 8.4. Excel implementation of the pulse-length optimiser routine showing optimisation of pulse-length for THF and water resonances with a Julia-optimised ν_0 (1690.27 Hz).

When implementing the calculated parameters into TopSpin, for one off resonance signals the offset can be inputted in the foreground using the spoffs[x] parameter. For multiple suppression sites, a phase ramp is imposed on an rectangular shape of optimal duration,$\boldsymbol{\tau}$, using $\boldsymbol{\Delta\nu}$ frequencies. This shape can be produced by the following TopSpin command (note that the Squa100.1000 shape will be overwritten). An example using parameters shown in Supplementary Fig. 8.5 is presented below:

st manipulate /opt/topspin4.1/exp/stan/nmr/lists/wave/user/Squa100.1000 offs e 68936 3 565.73 -580.27 -754.271/2

When simulated in ShapeTool for pulse length 68936 µs and $\gamma B_1/2\pi$ = 21.76 Hz (= 3*1/4*pw_{90}), the profile shown in Supplementary Fig. 8.5 was obtained. Note that changing the power level of the presaturation pulse has minimal influence on the level of suppression, stronger irradiation affects a wider range of signals.

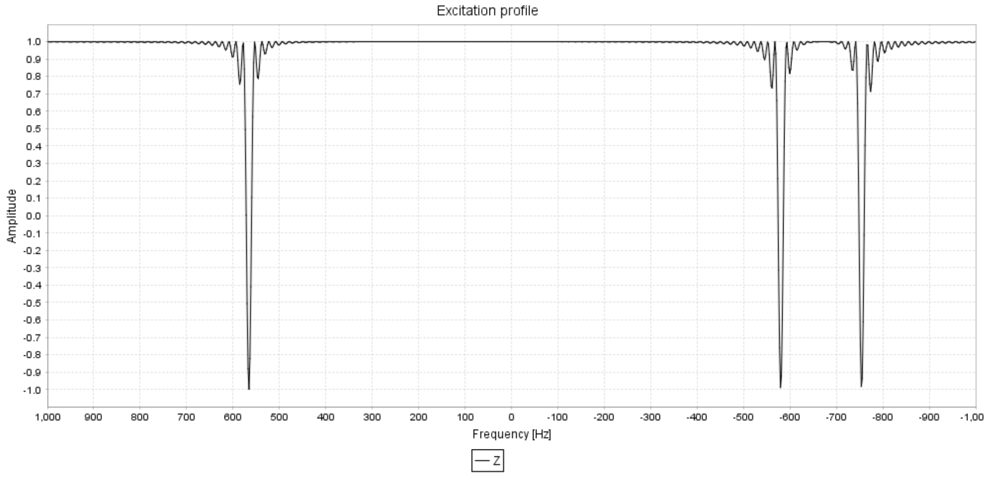

Supplementary Fig. 8.5. Inversion profile of a selective pulse affecting three resonance frequencies. Simulated using $\gamma B_1/2\pi$ = 21.76 Hz and resonance offsets 565.73 -580.27 -754.27 Hz relative to o1=1690.27 Hz. The affected regions show $\Delta_{1/2}$ = ~ 9Hz. Source data are provided as a Source Data Supplementary Fig. 8.5.xlsx.

Supplementary Note 9: 1D ^{1}H and SHARPER spectra of 2

9.1. Suppression of unwanted signals

Unwanted signals in the spectrum of **2** were suppressed by the pulse programme, **zgpr_pulse** (see Supplementary Software).

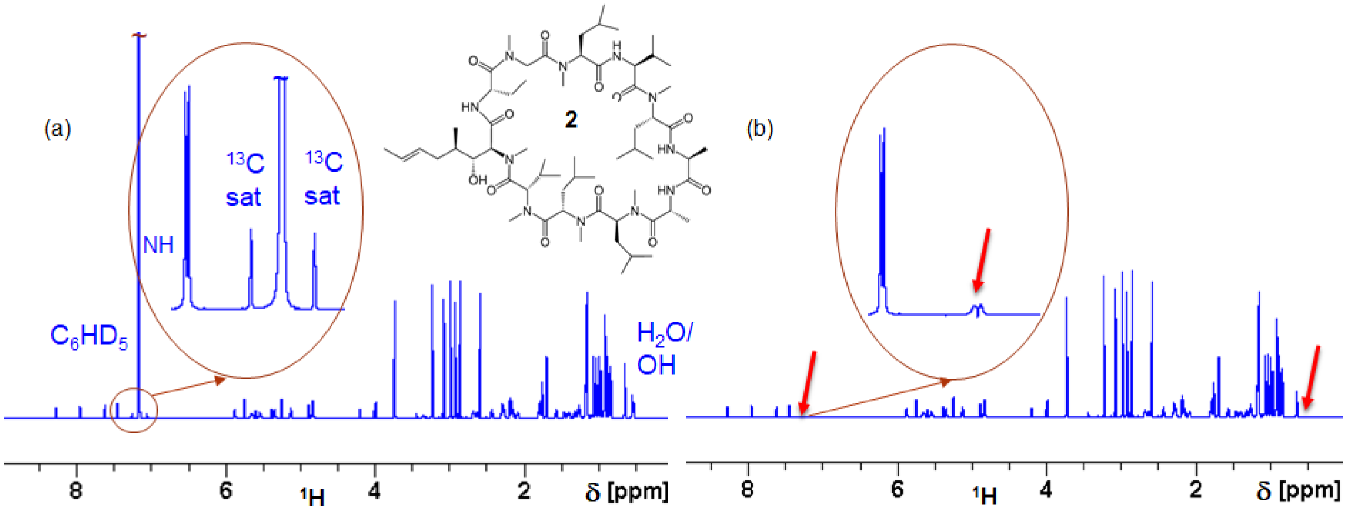

Supplementary Fig. 9.1. 800 MHz ^{1}H NMR spectra of **2** (a) 1D ^{1}H NMR spectrum; (b) spectrum acquired with a presaturation of the C_6HD_5 (main signals and the ^{13}C satellites) and H_2O/OH protons using the **zgpr_pulse** pulse seqeunce and parameters given in the Methods (main paper). The inset shows the structure of **2**.

9.2. Selecting the region to be collapsed

Aliphatic region of the spectrum of **2** was selected by using the pulse programme, **zgbspe** (see Supplementary Software).

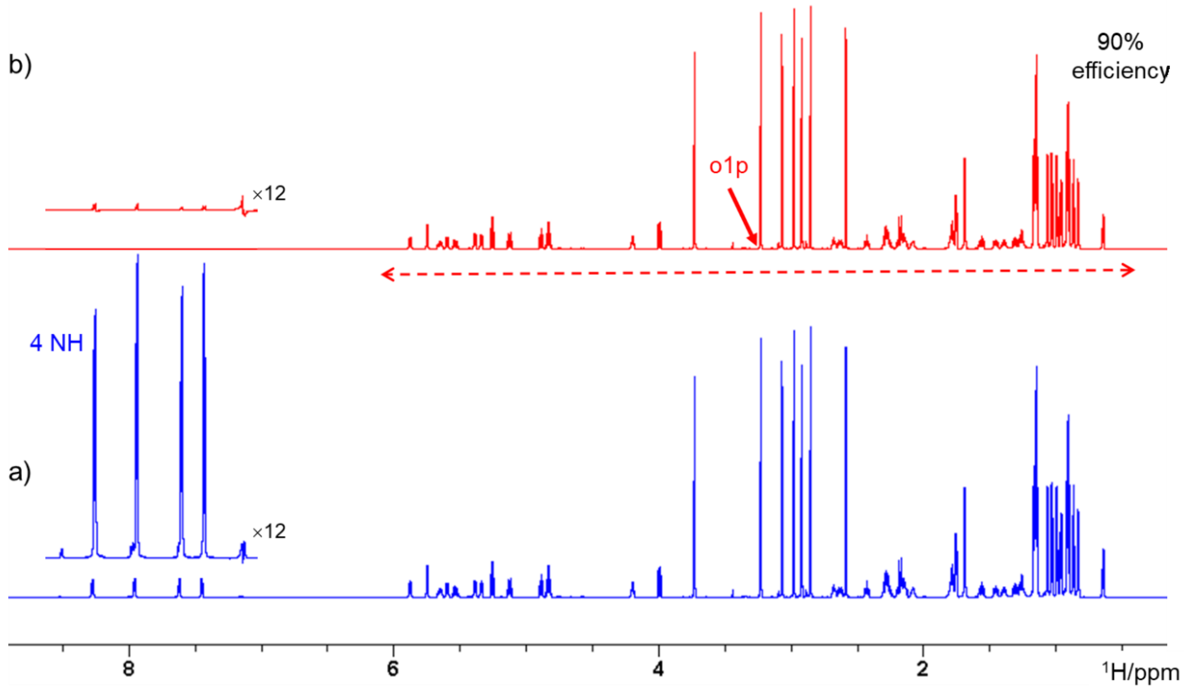

Supplementary Fig. 9.2. 800 MHz ^{1}H NMR spectra of **2**. (a) 1D ^{1}H spectrum; (b) 1D BSPE NMR spectrum acquired with a presaturation of the C_6HD_5 and H_2O/OH protons and ^{13}C decoupling (pulse sequence **zgbspe**). Insets show vertical expansions of the NH and the solvent region of spectra. Experimental parameters are given in the Methods (main paper).

9.3. 2D SHARPER-DOSY of 2

Prior to F_2 Fourier transformation of the 2D SHARPER-DOSY data, the imaginary time domain points were removed. Sixteen 1D DOSY traces acquired using 180° and 90° spin-echo pulses show very similar signal intensity with lower integral intensity but narrower signals for 90° pulses.

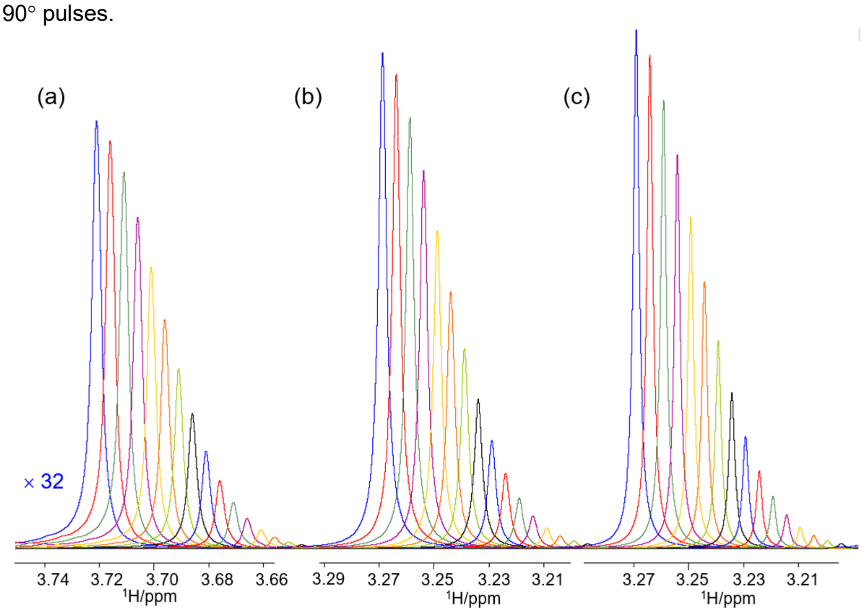

Supplementary Fig. 9.3. Decay of the DOSY signal of **2** using a 95 to 5% PFG ramp applied in 16 increments (a) the NCH_3 protons (3.722 ppm) from a regular DOSY spectrum scaled up 32 times; (b) and (c) the collapsed signal (3.35 ppm) from the SHARPER-DOSY acquired using 180° and 90° spin-echo pulse, respectively, and the pulse sequence of Fig. 5 of the main paper.

Supplementary Note 10: Time domain points or spectra? Analysis of diffusion coefficients.

The diffusion coefficients of sodium cholate in D_2O were evaluated at two different concentrations and by two methods. The time domain points of a series DOSY spectra acquired using increasing gradient strength are shown in Supplementary Fig. 11.1. Spectra of the dilute sample are shown in Fig. 8 of the main paper.

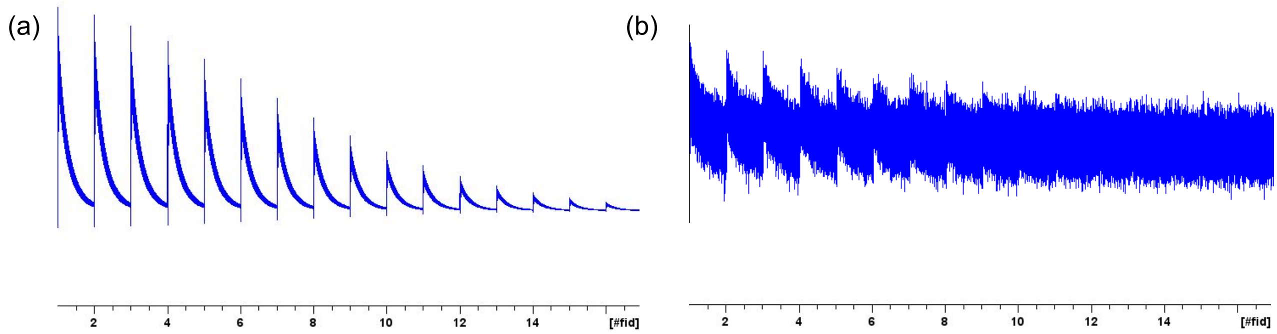

Supplementary Fig. 11.1. The real time domain points of 16 DOSY spectra of **3** (a) 7.7 mM and (b) 5.5 μM samples. The two figures are not to scale; (b) presents the time domain data after the subtraction of water impurities.

The results of the analysis are summarised in the Supplementary Table 1.

Supplementary Table 1.* Diffusion coefficient of **3** at two concentrations and two methods of data evaluation.

	Spectra integrated[(a)]	Time domain integrated[(d)]	
Concentration	Matched exponential line-broadening + FT	0-16,384 real points	Up to 1.26 T_2^S[(e)]
	Diffusion coefficient $D \times 10^9$ /m^{2}s^{-1}		
7.7 mM	0.353 ± 0.001[(b)]	0.353 ± 0.001	0.354 ± 0.001
5.5 μM	0.407 ± 0.003[(c)]	0.407 ± 0.003	0.387 ± 0.003

* Source data are provided as Source Data Supplementary Tables 1 and 2

[a] 16k of real data points were zero filled to 128k points and a matched exponential line broadening was applied (LB = 1.1 Hz and 0.78 Hz for the concentrated and dilute sample, respectively). Fifteen 1D DOSY spectra acquired as detailed in the Methods section of the main paper were fitted in Excel using Eqn 14.

$$\ln(I) = (\gamma_{^1H} g \delta)^2 \left(\Delta - \frac{\delta}{3}\right) D \qquad (14)$$

where *I* is the integral intensity, either of the SHARPER spectra or the time domain points, $\gamma_{^1H}$ is the gyromagnetic ratio of proton, g is the gradient amplitude, δ is the gradient length, Δ is the diffusion delay and *D* is the diffusion coefficient.

[b] Individual spectra had a signal-to-noise ratio (SNR) from 149,311:1 (1st spectrum) to 9636:1 (15th spectrum) and were integrated over the region of ± 2400 Hz to include the base of the SHARPER peak.

[c] Individual spectra had SNR from 135:1 (1st spectrum) to 6:1 (15th spectrum) and were integrated over the region of ± 16 Hz to include the base of the SHARPER peak. The 16th spectrum was not used becuse of poor SNR.

[d] Bruker time domain data were extracted using NMRglue (https://www.nmrglue.com/) and opened in Excell. Up to 16k of real data were integrated without any pre-processing by summing up their intensity.

[e] As the maximum SNR of the time domain data is obtained[5] for the acquisition time 1.26 T_2^*, the integration of the time domain data was stopped at this value. For the concentrated and the dilute sample this represented 6000 and 8000 points, respectively.

As seen in the Supplementary Table 1, a diffusion coefficient, $D_{7.7\,mM} = 0.353 \pm 0.001 \times 10^9$ /m^{2}s^{-1}, was obtained for the concentrated sample regardless of the evaluation method. For the dilute sample, an identical diffusion coefficient, $D_{5.5\,\mu M} = 0.407 \pm 0.003 \times 10^9$ /m^{2}s^{-1}, was obtained when the spectra or the time domain data points were integrated. However, the diffusion coefficient was different, $D_{5.5\,\mu M} = 0.387 \pm 0.003 \times 10^9$ /m^{2}s^{-1}, when the points were truncated at the 1.26 T_2^S.

To rationalise these results, our data were compared to those of Lindman *et al.*[6] The authors measured diffusion coefficients for a range of concentrations of the cholate anion in H_2O by an open-ended capillary tube method developed by Anderson and Saddington[7]. This data showed a linear dependency of the diffusion coefficient on concentration for solutions between 0.96 and 54 mM. After adjusting the published diffusion coefficients for D_2O as the solvent used in our experiments ($D_{D_2O} = D_{H_2O}/1.25$),[8] the following dependency of the diffusion coefficient on concentration of **3** for the specified range was obtained:

$$D_A = -(0.0036 \pm 0.002)c + 0.356 \pm 0.006 \qquad (15)$$

Using Eqn. 15, values were calculated for the concentrations used in our work and compared with the literature data (Supplementary Table 2).

Supplementary Table 2.* Comparison of measured diffusion coefficients of **3** with the literature data.[6]

Conc.	$D_A \times 10^9$ /m²s⁻¹ Based on Eqn. 15	$D_B \times 10^9$ /m²s⁻¹ All time domain points, or spectra	$D_C \times 10^9$ /m²s⁻¹ Time domain points up to 1.26 T_2^S	$\frac{D_A}{D_B}$	$\frac{D_A}{D_C}$
7.7 mM	0.332 ± 0.007	0.353 ± 0.001	0.353 ± 0.001	0.941	0.941
5.5 μM	0.360 ± 0.006	0.407 ± 0.003	0.387 ± 0.003	**0.886**	0.930

* Source data are provided as Source Data Supplementary Tables 1 and 2

For a 7.7 mM sample, this comparison shows a small difference (5.9 %) between the absolute values of the diffusion coefficients obtained by the open capillary method and NMR; the NMR values were independent of the data evaluation method. For the 5.5 μM sample this difference raised marginally to 7% when the time domain points were truncated at 1.26 T_2^S. However, when spectra or all time domain points were used, the difference increased to 11.4%.

The absolute differences between the open capillary and NMR methods can likely be attributed to systematic errors within either of the techniques. Nevertheless, the presented comparison indicates that for dilute samples integrating the initial time domain points leads to more accurate results than integrating all points or spectra.

The observed parity between the results obtained from spectra and all time domain points is not surprising as the two data sets are related by a mathematical operation, however, it should be noted that a matched exponential line broadening was only applied to spectra and not the time domain points. When integrating spectra, the results can be affected by noise within the integrated region, these therefore should be chosen carefully. Other possible processing approaches, i.e. apodising the time domain points prior to integration or applying stronger exponential line broadening prior to Fourier transformation of the data truncated at 1.26 T_2^S to avoid truncation artefacts were not explored.

In conclusions, based on this example, integration of real time domain points up to 1.26 T_2^S is recommended as a more accurate method for the determination of diffusion coefficients from DOSY data of dilute samples.

Supplementary references

1 Hurlimann, M. D. & Venkataramanan, L. Quantitative measurement of two-dimensional distribution functions of diffusion and relaxation in grossly inhomogeneous fields. *Journal of Magnetic Resonance* **157**, 31-42, doi:10.1006/jmre.2002.2567 (2002).

2 Jones, A. B., Lloyd-Jones, G. C. & Uhrin, D. SHARPER Reaction Monitoring: Generation of a Narrow Linewidth NMR Singlet, without X-Pulses, in an Inhomogeneous Magnetic Field. *Analytical Chemistry* **89**, 10013-10021, doi:10.1021/acs.analchem.7b02437 (2017).

3 Spencer, R. G. Equivalence of the Time-Domain Matched Filter and the Spectral-Domain Matched Filter in One-Dimensional NMR Spectroscopy. *Concepts in Magnetic Resonance Part A* **36A**, 255-265, doi:10.1002/cmr.a.20162 (2010).

4 Dickson, C. L., Peat, G., Rossetto, M., Halse, M. E. & Uhrin, D. SHARPER-enhanced benchtop NMR: improving SNR by removing couplings and approaching natural linewidths. *Chemical Communications* **58**, 5534-5537, doi:10.1039/d2cc01325h (2022).

5 Rovnyak, D., Hoch, J. C., Stern, A. S. & Wagner, G. Resolution and sensitivity of high field nuclear magnetic resonance spectroscopy. *Journal of Biomolecular NMR* **30**, 1-10, doi:10.1023/B:JNMR.0000042946.04002.19 (2004).

6 Lindman, B., Kamenka, N. & Brun, B. TRANSLATIONAL MOTION AND ASSOCIATION IN AQUEOUS SODIUM CHOLATE SOLUTIONS. *Journal of Colloid and Interface Science* **56**, 328-336, doi:10.1016/0021-9797(76)90258-7 (1976).

7 Anderson, J. S. & Saddington, K. S 80. The use of radioactive isotopes in the study of the diffusion of ions in solution. *Journal of the Chemical Society (Resumed)*, S381-S386 (1949).

8 Hardy, R. C. & Cottington, R. L. Viscosity of deuterium oxide and water in the range 5 to 125 C. *J. Res. Natl. Bur. Stand* **42**, 573 (1949).
