# Supplementary material for: SHARPER-DOSY: Sensitivity enhanced diffusion-ordered NMR spectroscopy
Source: Nat Commun. 2023 Jul 21;14:4410. doi: 10.1038/s41467-023-40130-2 (PMC10361965; doi:10.1038/s41467-023-40130-2)
Supplement: Supplementary file 3 — Supplementary Software [file 41467_2023_40130_MOESM3_ESM.zip › Supplementary Software/Supplementary software README file.docx]

**zim.py** –removal of the imaginary components of the 1D and 2D Bruker fid and ser files implemented as a python script

**Multi-reson-suppress.xlsx** – a multi-resonance signal suppression optimiser implemented in Excel

**PresatOptimise.jl** – optimisation of the duration of the selective pulse, $\tau$, and the carrier offset, $\nu_{0}$, written in the Julia programming language

**dosy_adsu** – subtracting the DOSY-SHARPER spectrum of the solvent from the DOSY-SHARPER of a sample using a Bruker AU program.

**Bruker pulse programs**

| **Name** | **Description** |
| --- | --- |
| zgpr_pulse | 1D sequence with signal presaturation using pulsed presat and optional ^13^C decoupling |
| zgbspe | Band selective excitation using perfect echo with 180° selective pulses. Incorporates signal presaturation by pulsed presat or changing frequencies and an optional ^13^C decoupling during presaturation |
| sharper_collapse | A pulse sequence to collapse all or some (selected by a band selective perfect echo) signals into a singlet.Incorporates signal presaturation by pulsed presat or changing frequencies and an optional ^13^C decoupling during presaturation |
| ledbpgp2s.compensated | A 2D pulse sequence for diffusion measurement using stimulated echo and LEDusing bipolar gradient pulses for diffusion, 2 spoil gradients and a compensating gradient. Incorporates an optional solvent presaturation using pulsed presat or changing frequencies during relaxation and diffusion delays |
| ledbpgp2s.sharper_collapse | A 2D pulse sequence for diffusion measurement using stimulated echo and LED based on Bruker's ledbpgp2s using 2 spoil gradients and a compensating gradient. Collapsing all or some signals (selected by a band selective perfect echo) into a singlet. Incorporates an optional solvent presaturation using pulsed presat or changing frequencies during relaxation and diffusion delays |
